# An incompressible state of a photo-excited electron gas

**DOI:** 10.1038/ncomms8210

**Published:** 2015-05-26

**Authors:** Alexei D. Chepelianskii, Masamitsu Watanabe, Kostyantyn Nasyedkin, Kimitoshi Kono, Denis Konstantinov

**Affiliations:** 1 LPS, Université Paris-Sud, CNRS, UMR 8502, Orsay F-91405, France; 2 Cavendish Laboratory, University of Cambridge, J J Thomson Avenue, Cambridge CB3 OHE, UK; 3 Low Temperature Physics Laboratory, RIKEN, Wako, Saitama 351-0198, Japan; 4 Quantum Condensed Phases Research Team, RIKEN CEMS, Wako, Saitama 351-0198, Japan; 5 Institute of Physics, National Chiao Tung University, Hsinchu 30010, Taiwan; 6 Institute of Physics, Kazan Federal University, Kazan 420008, Russia; 7 Okinawa Institute of Science and Technology, Onna, Okinawa 904-0412, Japan

## Abstract

Two-dimensional electrons in a magnetic field can form new states of matter characterized by topological properties and strong electronic correlations as displayed in the integer and fractional quantum Hall states. In these states, the electron liquid displays several spectacular characteristics, which manifest themselves in transport experiments with the quantization of the Hall resistance and a vanishing longitudinal conductivity or in thermodynamic equilibrium when the electron fluid becomes incompressible. Several experiments have reported that dissipationless transport can be achieved even at weak, non-quantizing magnetic fields when the electrons absorb photons at specific energies related to their cyclotron frequency. Here we perform compressibility measurements on electrons on liquid helium demonstrating the formation of an incompressible electronic state under these resonant excitation conditions. This new state provides a striking example of irradiation-induced self-organization in a quantum system.

## Abstract (web summary)

Two-dimensional phases of electrons exhibit interesting phenomena under magnetic fields. Chepelianskii *et al.* show that electrons on liquid helium exhibit an incompressible state when they are excited by a microwave field at particular frequencies related with the Landau level spacing.

## Graphical abstract

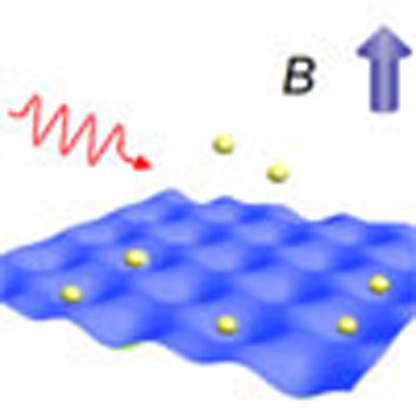

The discovery of the integer and fractional quantum Hall effects^1^,^2^,^3^,^4^,^5^,^6^ revealed the existence of new states of matter characterized by topological properties and strong electronic correlations triggering an intense theoretical and experimental research activity. These efforts lead to a detailed microscopic understanding of the main experimental phenomena and to some of the most beautiful conceptual breakthroughs in condensed matter physics^7^. The observation of a new dissipationless transport regime at low magnetic fields under microwave irradiation^8^,^9^ raised a new challenge regarding our understanding of two-dimensional electron systems. Microwave-induced zero-resistance states (ZRSs) appear at high-microwave excitation powers when the ratio between the photon energy *ħω* and Landau level spacing *ħω*_c_ is close to a value within the fraction series: *J*=*ω*/*ω*_c_=1+1/4,2+1/4,... In theory, however, the motion of electrons in a magnetic field in ultra-clean samples is well described as a harmonic oscillator where the selection rules only allow transitions between nearby oscillator states corresponding to *J*=1. The theoretical explanations proposed, so far, have attempted to resolve this contradiction by considering the role of sharp inhomogeneities due to a short-range disorder potential^10^,^11^,^12^, edges^13^,^14^ and contacts^15^. The dominant microscopic picture for ZRS is currently an ensemble of domains with vanishing local conductivity^12^ but the formation of a collective state with long-range order has also been suggested^16^,^17^. So far, the experimental evidence does not provide a definite proof in support of one of the available models and despite intense experimental efforts^18^,^19^,^20^,^21^,^22^,^23^,^24^ the microscopic nature of ZRS remains a puzzle. One of the difficulties is that while the manifestations of ZRS in transport phenomena are spectacular, such as vanishing longitudinal resistance, other indications of these novel electronic states remain elusive.

The observation of ZRS for surface electrons on helium under intersubband excitation^25^,^26^ has opened a new research direction in this field since the strong Coulomb interactions in this system allow collective effects, such as, for example, Wigner crystallization, to be observed more readily^27^,^28^. Previously, we reported a strong redistribution of the electron density under irradiation that coincided with the appearance of ZRS^29^, although the underlying mechanism was not elucidated. Since the formation of ZRS in a Hall system coincides with vanishing conductivity, the observed redistribution may simply be a consequence of the expected long charge relaxation rates in this regime. In the experiments presented here, we systematically study the behaviour of the electronic density under irradiation and demonstrate a regime in which electrons stabilize at a fixed steady-state density independent of their initial density profile and the electrostatic confinement potential. Since in this regime the electron density is not changed by an increase of the holding electrostatic forces, which tend to compress the electron cloud, we describe this new phase of the electron gas as an incompressible state.

## Results

### Description of the system

**Figure 1:**
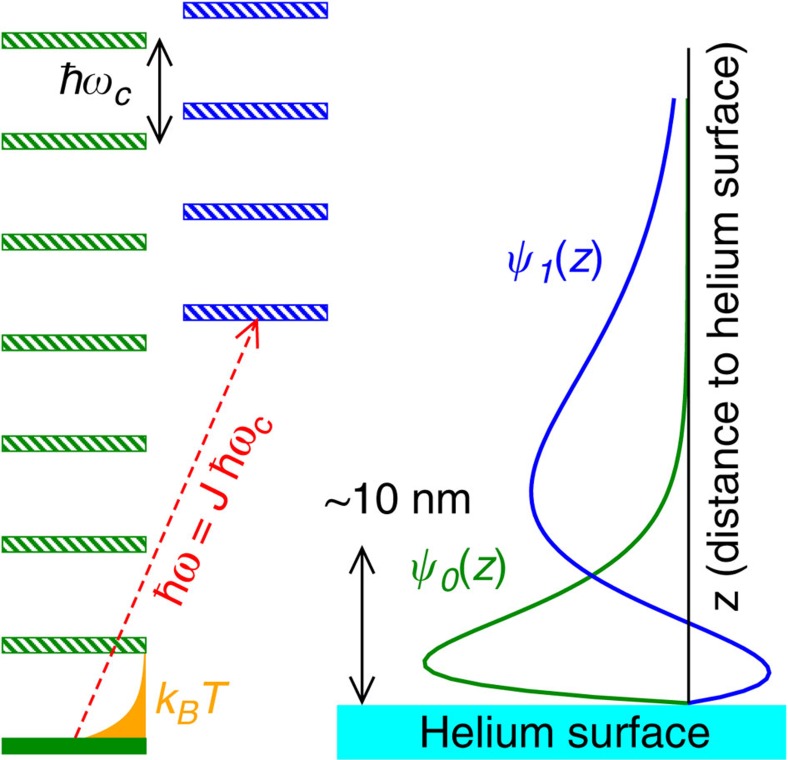
Accessible energy levels. Level diagram of the accessible states for the surface electrons in our experiment. Several bound states are formed owing to the attractive image charge potential created by the helium surface, the ground state and the first excited state are separated by energy *ħω*. Free motion along the helium surface transforms these states into conduction bands which under a quantizing magnetic field split into a manifold of discrete Landau levels. The ground state manifold is characterized by a wavefunction 
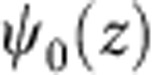
 that is localized close to the helium surface, whereas the wavefunction of the excited state manifold is centered farther away from the helium surface.

**Figure 2:**
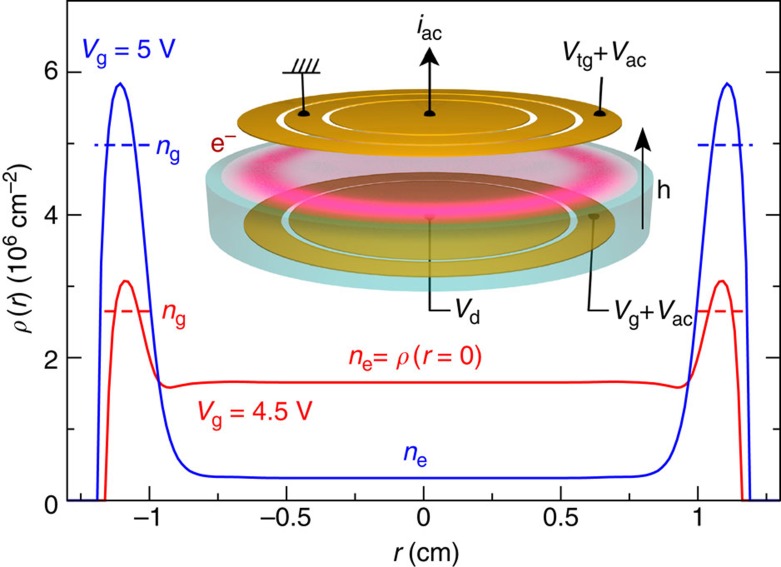
Electron distribution inside the cell. Theoretical electron density profiles *ρ*(*r*) for different values of the guard potential *V*_g_ for a cloud with *N*_e_=8 × 10^6^ trapped electrons and corresponding values of the mean electron density in the centre *n*_e_ and in the guard *n*_g_. The inset illustrates the helium cell geometry in our experiment and the electrostatic potentials applied to the electrodes during the compressibility experiments. The top of the cell is split in three circular electrodes with radii *R*_i_=0.7, *R*_d_=1, *R*_g_=1.3 cm, and the height of the cell is *h*=2.6 mm. For compressibility measurements, an a.c. driving potential is applied to both top and bottom guard electrodes and the induced image charge current *i*_a.c._ is measured on the central top electrode.

A ensemble of electrons was trapped on a liquid helium surface forming a non-degenerate two-dimensional electron gas. The energy levels accessible to the surface electrons in a quantizing perpendicular magnetic field are shown in Fig. 1. They are formed by Landau levels separated by energy *ħω*_c_ and intersubband excitations of energy *ħω* perpendicular to the helium surface^27^,^28^. Our experiments are performed at a temperature of *T*=300 mK much smaller than the Landau level spacing *k*_B_*T*≪*ħω*_c_ so that in equilibrium the electrons mainly fill the lowest Landau level, whereas under resonant irradiation at energy *ħω*, they can be excited into another subband manifold^25^,^26^. Note that the two-level system formed by the two subbands has been proposed as a candidate system for quantum computing^30^. Spatially, the electrons are distributed between the two regions on the helium surface (see Fig. 2), a central region above the disc-shaped electrode at potential *V*_d_ and a surrounding guard region above the ring electrode held at potential *V*_g_. We denote *n*_e_ and *n*_g_ as the mean electron densities in the central and guard regions, respectively. As in a field effect transistor, *n*_e_ and *n*_g_ can be controlled by changing the potentials *V*_d_ and *V*_g_. The key difference here is that for surface electrons the total number of electrons *N*_e_ in the cloud is fixed as long as the positive potentials *V*_d_ and *V*_g_ are sufficiently strong to balance the electron–electron Coulomb repulsion. Examples of simulated electron density profiles for different values of *V*_g_ are shown in Fig. 2, the simulations were performed within an electrostatic model as described in refs 31, 32, 33.

In addition to controlling the density profile of the electron cloud *ρ*(*r*), the potentials *V*_d_ and *V*_g_ also change the perpendicular holding field in the cell *E*_*z*_. To avoid this unwanted effect in our experiments, we fixed the value of *V*_d_ and changed the potential *V*_tg_ simultaneously with *V*_g_ keeping the difference *V*_tg_–*V*_g_ between top and bottom guard electrode voltages equal to *V*_d_. This choice ensures a uniform value of *E*_*z*_ across the cell. Since lowering the potential *V*_g_ compresses the electron cloud towards the centre of the helium cell, we define the compressibility of the electron system as *χ*=−*dn*_e_/*dV*_g_. In this definition, the electrostatic potential plays the role of the chemical potential in quantum Hall systems. This difference is due to the non-degenerate statistics for electrons on helium for which the Fermi energy is much smaller than the thermal energy.

### Compressibility in equilibrium

We developed the following method to measure the compressibility of the electron cloud. An a.c. voltage excitation with amplitude *V*_a.c._=25 m*V* was applied on the top and bottom guard electrodes (see Fig. 2) at a low frequency *f*_a.c._≃2 Hz, for which the electron density quasi-statically follows the driving potential. The induced modulation of *n*_e_ was measured by recording the a.c. current *i*_a.c._ created by the motion of image charges on the top central electrode with radius *R*_i_=0.7 cm. The correspondence between the variation of *n*_e_ and *i*_a.c._ was established using plane capacitance electrostatics, for which an electron trapped at the middle of the cell induces half an image charge of *e*/2 on the top electrode. The use of a simplified electrostatic model is justified here, since the gradients of *ρ*(*r*) are located away from the electrode on which *i*_a.c._ is measured. This leads to the following expression for the compressibility *χ*=−*dn*_e_/*dV*_g_:


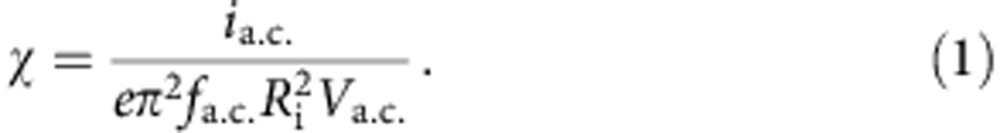


The presented measurement technique has a strong similarity to that used in compressibility experiments in the quantum Hall regime^34^, providing an additional justification for our definition of compressibility. In Supplementary Note 1, we provide a more thorough discussion on our definition of compressibility and give a detailed derivation of Equation 1, which is explained in Supplementary Note 2.

**Figure 3:**
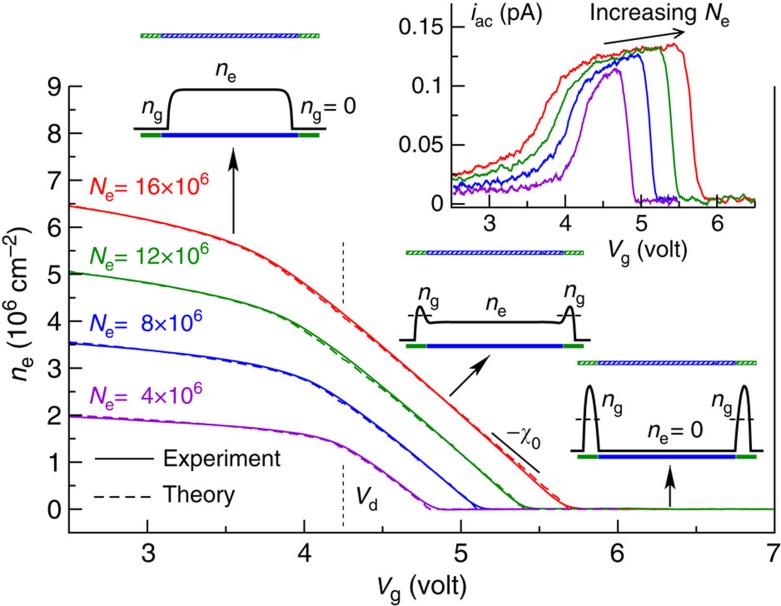
Density without irradiation. Dependence of *n*_e_ on the guard potential *V*_g_ without irradiation obtained using Equation 1 for different numbers of trapped electrons *N*_e_, the experimental values for the current are shown in the inset. The total number of electrons in the cloud is determined by comparing the measured dependence *n*_e_(*V*_g_) with theoretical calculations based on electrostatic modelling of the electron cloud. The sketches illustrate the typical electron density profiles along the *n*_e_(*V*_g_) curves. A very good agreement between experiment and modelling is achieved using the total electron number *N*_e_ as the only fitting parameter. Over a large range of *V*_g_ and *N*_e_, the compressibility is well approximated by the constant value χ_0_≃2.9 × 10^6^ cm^−2^ V^−1^. Temperature was *T*=300 mK, we verified that as expected these measurements were independent of the magnetic field.

Using Equation 1, the dependence *n*_e_(*V*_g_) can be reconstructed by integrating *χ* with respect to *V*_g_ starting from the high *V*_g_ limit where *n*_e_=0. The obtained results are illustrated in Fig. 3, which shows *i*_a.c._ and the corresponding density *n*_e_ as a function of *V*_g_ for several *N*_e_ values. The experimental curves for *n*_e_(*V*_g_) are compared with the results of electrostatic simulations^31^,^32^,^33^,^35^ that use the total electron number *N*_e_ as the single fitting parameter for each of the obtained curves. The simulations exhibit an extremely good agreement with the experimental results. The dependence *n*_e_(*V*_g_) can be understood as follows: a large positive potential *V*_g_ attracts the electrons towards the guard electrodes, whereas at low *V*_g_ the electrons are repelled from the guard region and concentrate at the centre of the cell. The potential from the guard electrodes is then almost completely screened, leading to a value of *n*_e_ that is almost independent of *V*_g_. At intermediate *V*_g_, electrons occupy both the central and guard regions. In this case, *n*_e_ decreases linearly with increasing *V*_g_ until the central region becomes completely depleted. At this point, *n*_e_=0 regardless of the value of *V*_g_. In the intermediate regime, the compressibility *χ*=−(*dn*_e_)/(*dV*_g_) depends only weakly on the total number of trapped electrons *N*_e_ and the values of the confining potentials. This observation can be understood by considering a simplified electrostatic model in which the two electron reservoirs in the disc and guard are treated as plane capacitors. This model, which is presented in more detail in Supplementary Note 3, leads to a value for *χ* that depends only on the geometrical cell parameters (provided the disc/guard reservoirs are not empty):


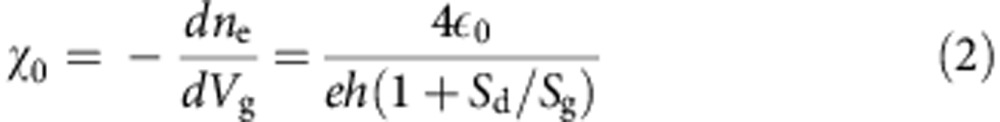


Here, *h*=2.6 mm is the cell height, 
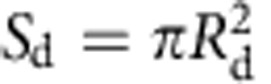
 and 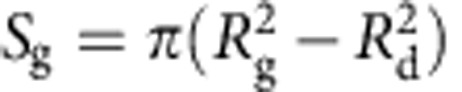 are the surface areas of the bottom disc/guard electrodes (*R*_g_ being the outer radius of the guard electrodes) and 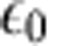 is the vacuum permittivity. We experimentally find χ_0_≃2.9 × 10^6^ cm^−2^ V^−1^ in good agreement with the estimation obtained using the geometrical cell parameters *R*_d_=1 cm and *R*_g_=1.3 cm. This reference value will be used to normalize the compressibility in our following experiments.

### Compressibility under irradiation

We next present our compressibility measurements in the presence of microwave irradiation, focusing on the ZRS fraction *J*=*ω*/*ω*_c_=6.25, with *ω*=2*π* × 139 GHz and a magnetic field of *B*=0.79 T. Outside ZRS regions the compressibility is not changed by microwave irradiation since it is independent of the conductivity of the system *σ*_xx_ provided it remains finite. The holding field, identical in the central and guard regions (*V*_d_=*V*_g_–*V*_tg_=4.24 V), was chosen to tune the photon energy in resonance with the intersubband transition.

**Figure 4:**
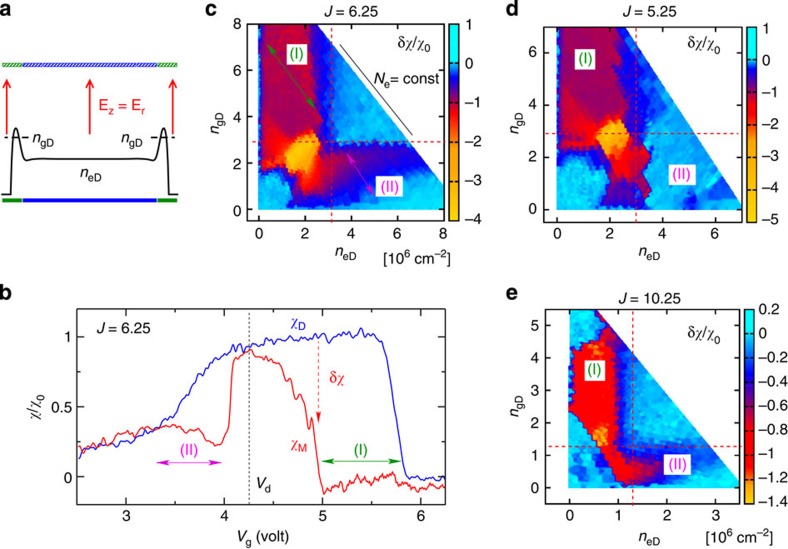
Compressibility under irradiation. Compressibility *χ* as a function of guard voltage *V*_g_ in the dark (*χ*_d_) and under microwave irradiation (*χ*_*M*_) at a frequency of *ω*=2*π* × 139 GHz and a magnetic field *B*=0.79 T corresponding to *J*=*ω*/*ω*_c_=6.25. As shown in (**a**), the perpendicular electric field was fixed to the intersubband resonance value *E*_*r*_ in both the central and guard regions. (**b**) The compressibility under microwave irradiation for *N*_e_=18 × 10^6^, *χ* changes under irradiation in two distinct regions: region (*I*), where *χ*_*M*_ almost vanishes under irradiation and region (*II*), where *χ*_*M*_ is significantly reduced. (**c**) *δχ*/*χ*_0_ measured at different *N*_e_ values as a function of the equilibrium density at the centre *n*_eD_ and in the guard *n*_gD_. The boundaries of the anomalous regions (*I*) and (*II*) are aligned along lines of constant density *n*_eD_ and *n*_gD_, respectively. The colour scale indicates the change in the compressibility under irradiation, the value *δχ*/*χ*_0_=−1 (red colour) corresponding to incompressible states. The positions of the coloured arrows corresponds to the trace in **b**. (**d**,**e**) Results similar to **c** but obtained in two other ZRS regions: *J*=5.25 and *J*=10.25. The vertical/horizontal boundaries coincide approximately at *J*=5.25 and *J*=6.25 but are located at significantly lower densities for *J*=10.25.

The compressibilities measured in the dark *χ*_d_ and under irradiation *χ*_*M*_ are compared in Fig. 4. Two singular regions where *χ*_*M*_ and *χ*_d_ differ are present: region (*I*) at high *V*_g_ and region (*II*) at low *V*_g_. Hereafter on we use different notations for the average densities in the dark *n*_eD_ and *n*_gD_ and under microwave irradiation *n*_eM_ and *n*_gM_ to avoid ambiguity. In region (*I*), the dark density in the guard is higher than at the centre *n*_gD_>*n*_eD_, whereas in region (*II*) we have *n*_gD_<*n*_eD_. In both regions, a strong suppression of the compressibility is observed and *χ*_*M*_ strikingly vanishes in most of region (*I*). In contrast, at *V*_g_=*V*_d_=4.24 V where the electrons are distributed evenly between centre and the guard, the compressibility remains almost unchanged under irradiation: *χ*_*M*_≃*χ*_d_≃*χ*_0_.

To clarify the physical origin of the anomalous regions (*I*) and (*II*), we convert the experimentally controlled variables *V*_g_ and *N*_e_ to physically more relevant densities *n*_eD_ and *n*_gD_. We obtained *n*_eD_ and *N*_e_ from the compressibility measurements in the dark (as in Fig. 3). The quantity *n*_gD_ could not be measured directly in a reliable manner owing to the unavoidable effect of the density gradients in the guard region. We, thus, calculated *n*_gD_ by averaging the simulated density profiles *ρ*(*r*) over the guard region. This procedure is justified by the excellent agreement between the compressibility measurements and our numerical simulations that we demonstrated in absence of irradiation.

Using this method, we summarize on the *n*_eD_,*n*_gD_ plane the changes in compressibility *δχ*=*χ*_*M*_–*χ*_d_ under irradiation measured at different values of *V*_g_ and *N*_e_ while fixing all the other parameters (magnetic field, microwave frequency and power, temperature and perpendicular electric field; the dependence on microwave power and magnetic field is shown in Supplementary Figs 1–5). The results are shown in the colour-scale panels in Fig. 4. The anomalous regions (*I*) and (*II*) are upper bounded by lines of constant density in the centre *n*_eD_=*n*_c_ and in the guard *n*_gD_=*n*_c_ where we introduced *n*_c_≃3 × 10^6^ cm^−2^. Indeed, for *n*_gD_>*n*_c_ and *n*_eD_>*n*_c_ the change in compressibility |*δχ*|≪*χ*_0_ is negligible and the electron density is still described by the electrostatics of the gas phase. Similarly, at low densities: *n*_eD_ and *n*_gD_<1.5 × 10^6^ cm^−2^, we also find no deviations from the equilibrium *χ* values. The incompressible regions (*I*) and (*II*) occupy the space *n*_eD_<*n*_c_<*n*_gD_ and a fraction of the space *n*_gD_<*n*_c_<*n*_eD_, they are characterized by *δχ*/*χ*_0_≃−1 in Fig. 4. Finally, in the remaining area on the *n*_eD_,*n*_gD_ plane, *χ* becomes negative under irradiation, for example, at *n*_eD_=*n*_gD_=2 × 10^6^ cm^−2^. To highlight the robustness of our results, Fig. 4 also shows similar data obtained at two other ZRS fractions: *J*=5.25 and *J*=10.25. Incompressible regions appear for these cases as well, we note that for *J*=10.25 the position of the boundary *n*_c_ is displaced towards significantly lower densities *n*_c_≃1.3 × 10^6^ cm^−2^.

The incompressible regions with vanishing *χ* correspond to an unexpected regime where the density becomes independent of the compressing confinement potential *V*_g_. In the integer quantum Hall effect, incompressible phases appear owing to the finite energy required to add electrons to a system in which the Landau levels available at the Fermi energy are all fully occupied. This explanation, however, is not applicable to electrons on helium since they form a non-degenerate electron gas. Experiments on the quantum Hall effect have also shown that a vanishing longitudinal conductivity *σ*_xx_, can freeze a non-equilibrium electron density distribution since the charge relaxation time scales can become exponentially large^36^,^37^. An explanation based on the vanishing conductivity *σ*_xx_ seems natural owning to the coincidence between the onset of charge redistribution and ZRS. We can determine experimentally whether the incompressible behaviour can be explained only on the basis of *σ*_xx_=0. Indeed, a state with *σ*_xx_=0 is expected to freeze the existing density distribution due to the very long charge relaxation rates; thus, the final state should depend in a non-trivial way on the equilibrium density profile and on the kinetics of the transition to ZRS.

We developed the following approach to determine the central density under irradiation *n*_eM_ for different initial densities *n*_eD_. We performed compressibility measurements without irradiation as described in Fig. 3 to obtain *n*_eD_ as function of *V*_g_ at a fixed *N*_e_. Then, fixing *V*_g_, we irradiated the electron system with on/off pulses of millimetre waves creating a periodic displacement of the electron density *δn*_e_=*n*_eM_–*n*_eD_. This displacement induces a transient current of image charges on the measuring electrode *i*_pv_(*t*), which within a plane capacitor approximation is related to the change in the electron density *δn*_e_ through the relation:


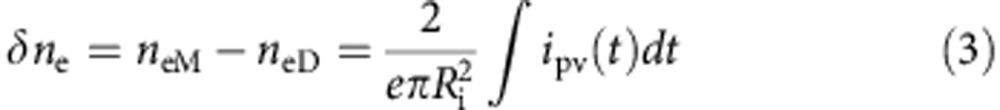


**Figure 5:**
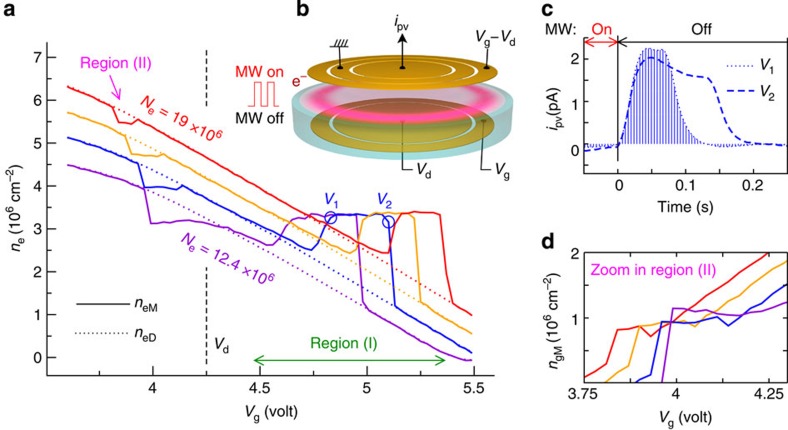
Photocurrent density measurements. (**a**) Density under irradiation as determined from transient photocurrent measurements. The electron cloud was prepared at different initial density distributions by changing *V*_g_ and performing experiments at several values of *N*_e_=12.4,14.5,16.8,19 [ × 10^6^]. The dependence of the equilibrium density *n*_eD_ on *V*_g_ was determined using the method from Fig. 2, it is indicated by dotted lines. For each initial condition, we then applied a sequence of on/off microwave pulses and recorded the photocurrent *i*_pv_ induced by the electron redistribution. The measurement geometry is shown in **b**. Two *i*_pv_(*t*) traces are shown in **c** for two *V*_g_ values noted *V*_1_=4.83 V and *V*_2_=5.1 V at *N*_e_=14.5 × 10^6^. The change in the density *δn*_e_ due to irradiation was then determined from Equation 3, it is proportional to the area below the *i*_pv_(*t*) curves (shaded region for *V*_g_=*V*_1_). Region (*I*) shows a clear plateau at *n*_eM_≃3.5 × 10^6^ cm^−2^, where the density is independent of the initial density distribution. (**d**) A magnification of region (*II*) shows the density in the guard under irradiation, determined approximatively from *n*_gM_=(*N*_e_–*n*_eM_*S*_d_)/*S*_g_.

The integral in this equation is evaluated over the time interval where the irradiation is switched off (a derivation of this relation is provided in Supplementary Note 4). Combining *δn*_e_ with the known values for *n*_eD_, we reconstructed the dependence of *n*_eM_ on the guard potential *V*_g_: the results are shown oi Fig. 5 for several *N*_e_ values.

Deviations from *n*_eD_ mainly appear in two voltage regions, which are in good correspondence with the regions (*I*) and (*II*) outlined in Fig. 4. In region (*I*), the density under irradiation exhibits a striking plateau as a function of *V*_g_ with a plateau density independent of *N*_e_. We emphasize that this plateau appears because of the cancellation between the decrease of *n*_eD_ at higher *V*_g_ and the increase of the area below *i*_pv_(*t*) curves (see Fig. 5c and Equation 3), it is thus a highly non-trivial experimental result. These observations confirm the existence of an incompressible phase for electrons on helium and appear to exclude an explanation based only on *σ*_xx_=0 since the final state density does not depend on the density distribution in equilibrium for a wide range of parameters. Instead, our experiments imply the existence of a dynamical mechanism that stabilize the electron density to a fixed value.

We next comment on the sign of *δn*_e_. In region (*I*), the electrons migrate from the guard, where the densities are higher, to the centre of the electron cloud, whereas in region (*II*), the trend is opposite and electrons flow from the centre towards the edges of the electron cloud. In the latter region, narrow density plateaux are also observed, however, the plateau density value depends on *N*_e_ in contrast to region (*I*). An approximate calculation of *n*_gM_ shown in Fig. 5 suggests that in region (*II*), the transition to an incompressible state occurs owning to the pinning of the density inside the guard region. Thus, in the incompressible phase, the electron cloud transfers electrons from a high-density reservoir region, increasing the density in the low-density regions up to a plateau value.

## Discussion

Since the formation of a non-equilibrium density profile increases the electron–electron repulsion energy, it is important to estimate the associated energy cost. Using the simplified electrostatic model (see Supplementary Note 5 for a detailed derivation), we find that the energy cost of the redistribution per electron Δ_e_ is approximately:


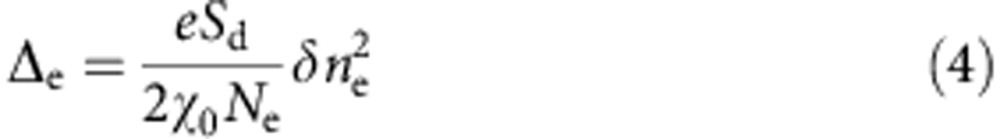


From the experimental values of *δn*_e_=1.5 × 10^6^ cm^−2^ and *N*_e_=12.4 × 10^6^ (estimated at point *V*_2_ in Fig. 5), we find Δ_e_≃0.1 eV. The presence of this large electrostatic barrier can explain why the incompressible regions occupy a narrower *V*_g_ range in the photocurrent data than in the compressibility measurement: for example, region (*I*) has a width at least 0.6 V in Fig. 4 but a width of only 0.3 V in Fig. 5. The main difference between the two techniques is that during compressibility measurements microwaves are always present, maintaining the system in a non-equilibrium state, whereas in the photocurrent measurement the microwave on/off pulses continuously reset the system back to its equilibrium state. Thus, in the photocurrent measurements, the electrons must overcome an increasingly large energy barrier to reach the incompressible state as *V*_g_ increases. When the barrier becomes too large, the systems remains in its equilibrium state and the photocurrent vanishes abruptly. In contrast, in the compressibility measurement, the electron system remains in the incompressible state as *V*_g_ changes and the electrostatic barrier does not need to be overcome directly, allowing the incompressible state to exist over a wider parameter range. (In the Supplementary Figs 6–9, we provide a detailed comparison between the two measurement techniques and show that they are fully consistent once the described hysteretic behaviour is taken into account; additional experimental data is also provided in ref. 38).

The estimated charging energy must be provided by the microwave irradiation since it is the only energy source in our system, its amplitude corresponds to a surprisingly large number of photons absorbed per electron Δ_e_/*ħω*≃170, particularly in comparison with a two-level system that cannot absorb more than one photon. The energy of the absorbed photons thus needs to be transferred efficiently to other degrees of freedom. It could be transferred by the excitation of higher Landau levels. However, the theoretical calculations performed by Y. Monarkha to explain the origin of microwave-induced resistance oscillations for electrons on helium suggest that Landau levels higher than the photon energy are unlikely to be strongly populated^39^,^40^. These calculations give an accurate prediction for the phase of the resistance oscillations allowing us to exclude strong inter-Landau level excitation^41^.

The energy can also be accumulated by transitions within the same manifold of quasi-degenerate Landau levels as they are bent by the confinement potential. A possible mechanism for this absorption is provided by the negative conductivity models introduced theoretically to explain ZRS in heterostructures^12^. It has been predicted that a negative resistance state will stabilize through the formation of domains with a fixed built-in electric field *E*_c_. We performed simulations of the electron density profiles for an electric field dependent conductivity model with *σ*(*E*)∝(*E*^2^−*E*_c_^2^), which is believed to describe domain formation. Our simulations show that this model tends to fix the electron density gradients to generate the built-in field *E*_c_. This changes the compressibility of the electron cloud depending on the number of stable domains in the system but without inducing an incompressible state. Thus, new theoretical developments are required to make the domain picture consistent with our experiments.

A recent theoretical proposal suggests that another instability can occur even before the conductivity becomes negative^42^,^43^. Due to fluctuations of the microwave electromagnetic field on the wavelength scale *λ*≃2 mm ratchet internal currents are expected to appear under irradiation inside the electron system. The velocity of the induced electron flow will scale as 
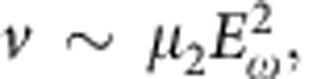
 where *E*_*ω*_ is the amplitude of the microwave electric field and *μ*_2_ a non-linear response coefficient^43^. Under steady-state conditions, this internal flow must be compensated by a counter-flow created by internal electric fields with maximal value 
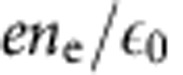
. The balance between the two flows sets a lower bound on the longitudinal mobility that is *μ*_xx_ stable under irradiation: 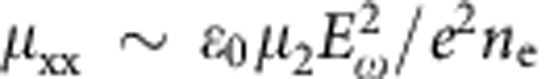. Mobilities below this threshold are expected to lead to the formation of electron pockets on a scale *λ* with a certain similarly to the stripe/puddles instability occurring in quantum Hall systems^44^.

The above argument predicts the existence of an instability without explaining the pinning of the electron density. A mechanism selecting a particular density value is thus desirable. As a possible mechanism, we propose the following scenario. If we assume that a significant fraction of the energy carried by the absorbed photons is transferred into ripplons with a typical wave number given by the inverse magnetic length 
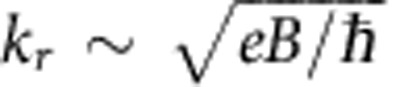
 (ref. 45), then the helium surface will vibrate at frequency 
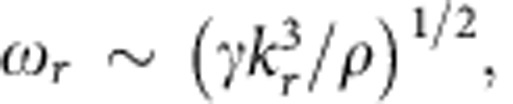
 where *γ* and *ρ* are the helium surface tension and density, respectively^28^. Provided frequencies are matched, this vibration can interact resonantly with electronic modes. The frequency of the expected surface vibrations is *ω*_*r*_≃2π × 30 MHz (for *B*=0.79 T) and has the same order of magnitude as the low wavelength magneto-shear modes^46^, which have frequency 
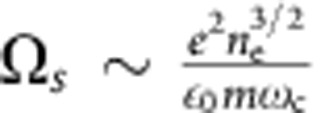
. Equating these frequencies *ω*_*r*_∼Ω_*s*_, we find an equation on the plateau density:


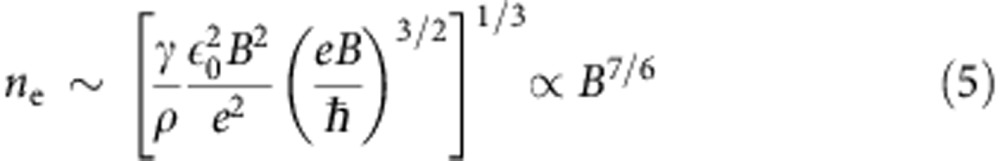


For *B*=0.79 T, this formula gives the right order of magnitude for the plateau density *n*_*e*_≃5.8 × 10^6^ cm^−2^, and the predicted dependence on *B* is consistent with our observations at different *J*=*ω*/*ω*_c_ shown in Fig. 4. Even if the described mechanism requires further theoretical studies, it correctly captures the critical dependence on the electronic density observed in the experiments. For electrons on helium, the electronic density mainly controls the strength of electron–electron interactions and the key role played by this parameter indicates that the formation of incompressible states is a collective effect involving many electrons.

In conclusion, we have shown that electrons trapped on a helium surface exhibit incompressible behaviour under resonant irradiation conditions corresponding to the formation of a microwave-induced ZRS. Their density becomes pinned to a fixed value independent of the applied electrostatic force and on the initial electron distribution profile for a wide range of parameters. The transition to the incompressible state is achieved by overcoming an impressively high electrostatic energy barrier of up to 0.1 eV per electron. We described the possible energy conversion processes within the electron system that can transform the energy of the absorbed photons into charging energy, and we proposed several competing mechanisms that can render the equilibrium density profile unstable. Since the incompressible behaviour emerging from our experiments is very elegant, we believe that its understanding will stimulate the emergence of new concepts for self-organization in quantum systems.

## Methods

Our experiments were performed in a Leiden dilution refrigerator with base temperature around 25 mK, the magnetic field was provided by a homemade superconducting magnet with maximal field of 1 T. The experimental cell was filled with liquid helium until half filling by monitoring the cell capacitance during helium condensation. Electrons were deposited on the helium surface by thermionic emission from a heated tungsten filament. Control of the total electron number *N*_e_ in the cloud was achieved by trapping an initially high concentration of electrons and then lowering the confinement voltages allowing excess electrons to escape. The obtained *N*_e_ value was determined from compressibility measurements in equilibrium. The frequency of the intersubband transition was tuned in resonance with the photon energy using the linear Starck shift induced by the electric field perpendicular to the helium surface, which was fixed during our compressibility and photocurrent experiments to keep the system at intersubband resonance. The current probe electrodes were grounded through Stanford Research (SR570) current amplifiers, while the potential of the other electrodes was set by Yokogawa DC voltage supplies, this ensure a stable d.c. voltage on all cell electrodes independently of the irradiation. Numerical simulations were performed by solving the Laplace equations for our cell geometry using a finite elements method^47^.

## Additional information

**How to cite this article**: Chepelianskii, A.D. *et al.* An incompressible state of a photo-excited electron gas. *Nat. Commun.* 6:7210 doi: 10.1038/ncomms8210 (2015).

## Supplementary Material

Supplementary Information Supplementary Figures 1-9, Supplementary Notes 1-5 and Supplementary References
