# Supplementary material for: An incompressible state of a photo-excited electron gas
Source: Nat Commun. 2015 May 26;6:7210. doi: 10.1038/ncomms8210 (PMC4455090; doi:10.1038/ncomms8210)
Supplement: Supplementary Information — Supplementary Figures 1-9, Supplementary Notes 1-5 and Supplementary References [file ncomms8210-s1.pdf]

Supplementary Figure 1. Dependence on microwave power

As demonstrated in Supplementary Figure 1 the microwave power changes the surface of the incompressible regions on the $(n_{\rm eD}, n_{\rm gD})$ plane without changing their horizontal/vertical boundaries. The observation that the $n_{\rm eD}$ and $n_{\rm gD}$ density boundaries are independent on the microwave power supports our idea of a resonance that occurs around certain electron densities independently of the microwave power. At lowest microwave power the narrow incompressible region clearly follows lines of constant $N_{\rm e}$. This can be understood from the hysteretic behavior which we described in the main manuscript. At lowest microwave powers, the incompressible state can only form within a narrow range of $N_{\rm e}$ values. Once the incompressible state is formed, it can exist as a meta-stable state over a wide range of guard voltages; which when converted into the corresponding dark density parameters $n_{\rm gD}$ and $n_{\rm eD}$ give a narrow incompressible stripe tilted along the slope $N_{\rm e} = {\rm const}$.

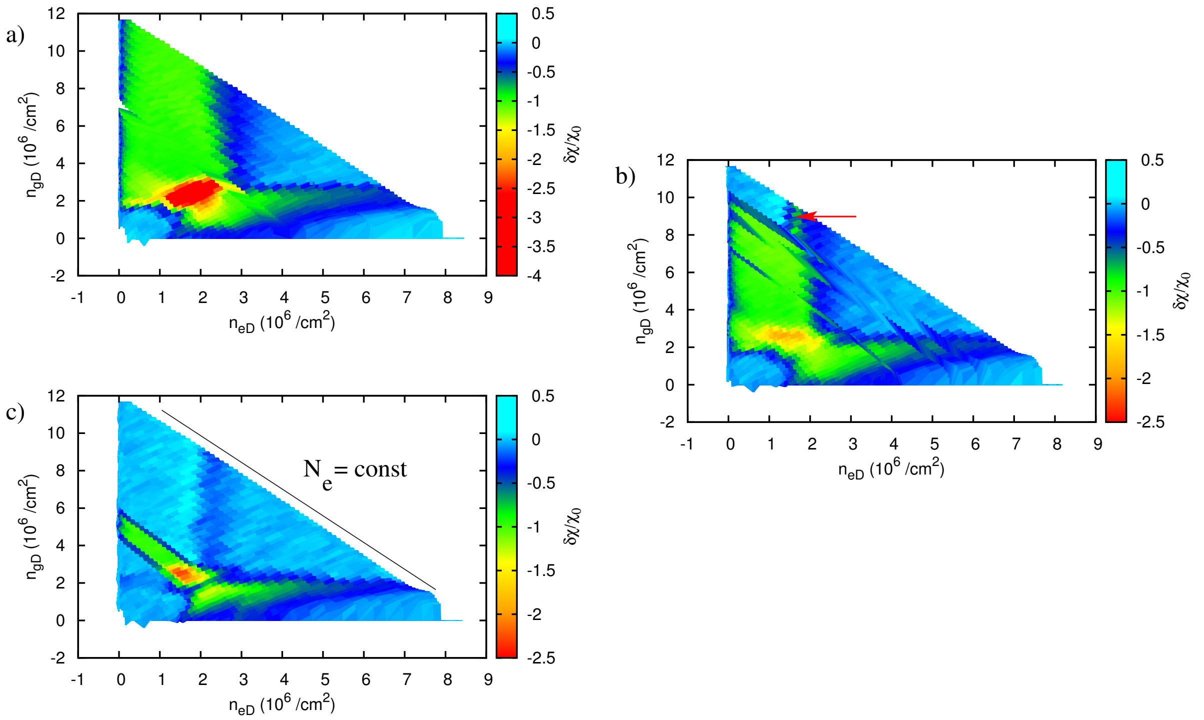

Supp. Fig. 1: Evolution of the incompressible regions (colored in green) as a function of the dark densities $n_{\rm eD}$ and $n_{\rm gD}$ for different microwave powers at $J = 6.25$. Panel a) corresponds to the maximal microwave power and is identical to Fig. 4 in the main manuscript but with a different color-code. In panel b) the microwave power was reduced to half of its maximal value, and in panel c) the microwave power was 30% of the maximum. The red-arrow on panel b) indicates the peak in $\delta\chi/\chi_0$ just outside the incompressible region supporting our suggestion of a density dependent resonance. The explicit dependence on $N_{\rm e}$ supports the existence of long-range correlations in the system since not only the local densities are important.

Supplementary Figure 2. Magnetic field dependence around $J = n + 1/4$ (n integer).
We did not observe any strong dependence on the parameter $J = \omega/\omega_c$ as long as its value was near a minimum of the MIRO oscillations (integer plus 1/4 offset), the corresponding data is shown in supplementary figure 2. When the deviation of J became too large the incompressible states disappeared abruptly and the equilibrium dependence $n_e(V_g)$ was recovered. This shows that the integer part of J is the important parameter for the study of the magnetic field dependence of the incompressible states.

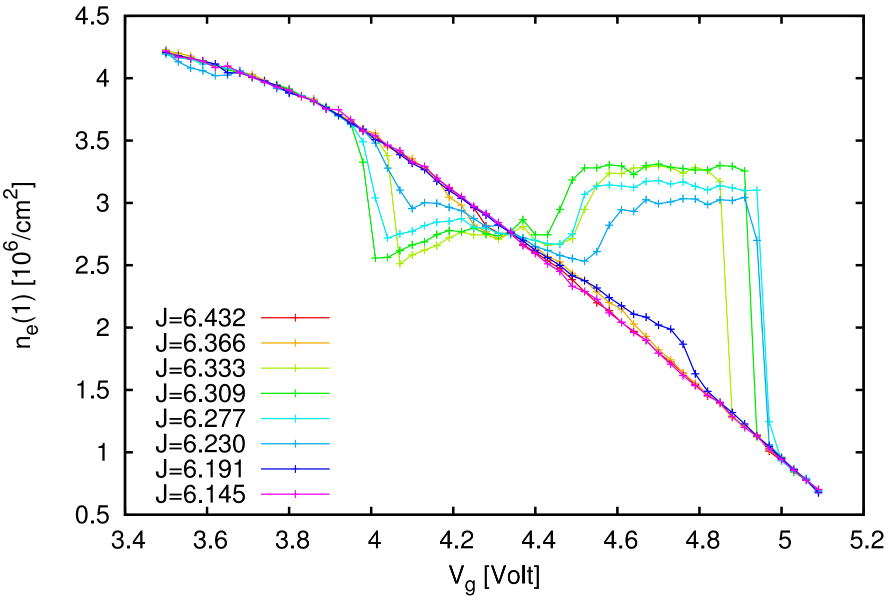

Supp. Fig. 2: Density under microwave irradiation n_{eM} obtained from the photo-current technique as a function of V_g for different values of $J = \omega/\omega_c$ around $J \simeq 6.25$. The experimental protocol is identical to Fig. 5 from the main manuscript. The total number of electrons in the cloud during this experiment was estimated to be: $N_e \simeq 11.5 \times 10^6$. Incompressible plateaux only appear for $J \in (6.23, 6.333)$, outside this interval values very close to n_{eD} are recovered.

Supplementary Figure 3: Compressibility at $J = 10.25$

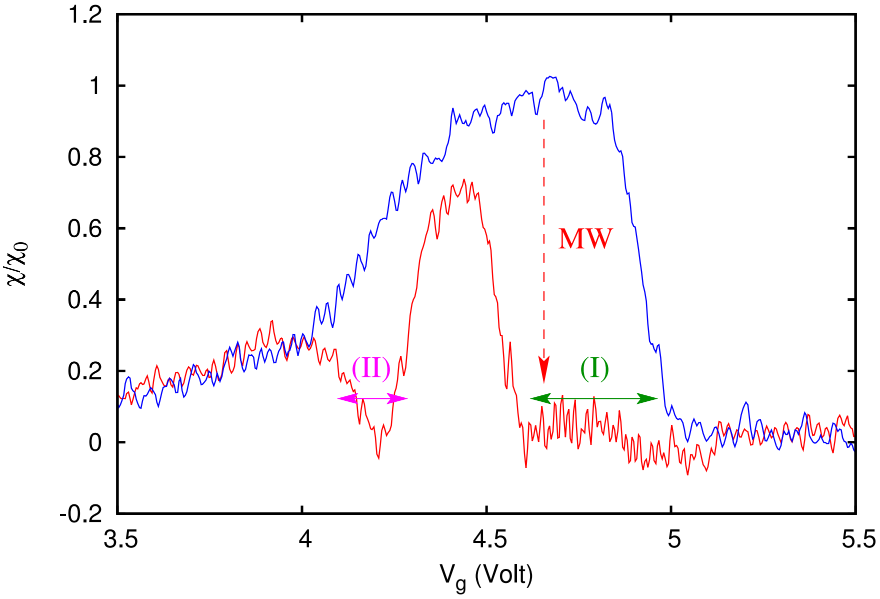

Supp. Fig. 3: Compressibility in equilibrium (blue) and under microwave irradiation (red) for $N_\mathrm{e} = 7.2 \times 10^6$ at $J = 10.25$. The structure is very similar to Fig. 4 (main text) with the appearance of two distinct incompressible regions.

Supplementary Figure 4: Compressibility at $J = 5.25$

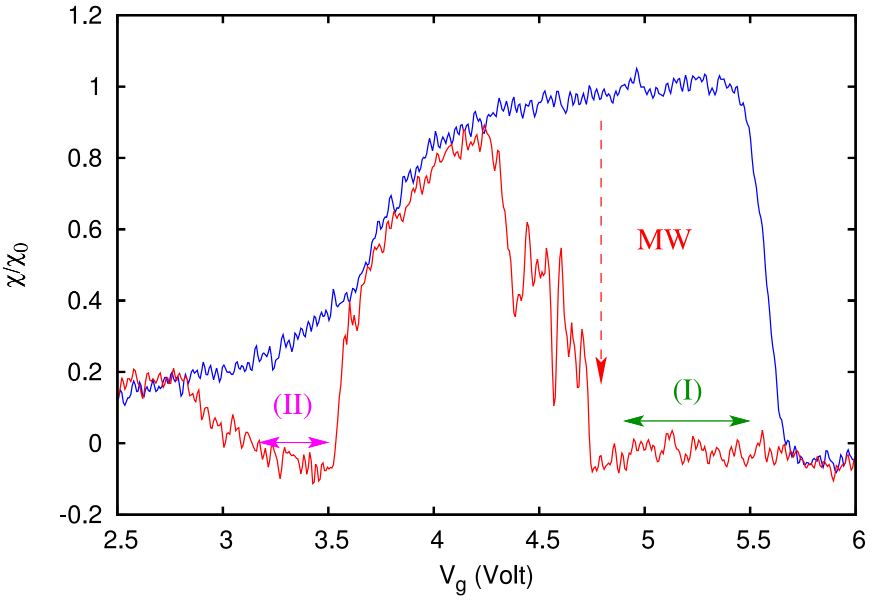

Supp. Fig. 4: Compressibility in equilibrium (blue) and under microwave irradiation (red) for $N_{\mathrm{e}} = 14 \times 10^6$ at $J = 5.25$. The structure is very similar to Fig. 4 (main text) with the appearance of two distinct incompressible regions.

Supplementary Figure 5: Density from the photocurrent technique at $J = 5.25$

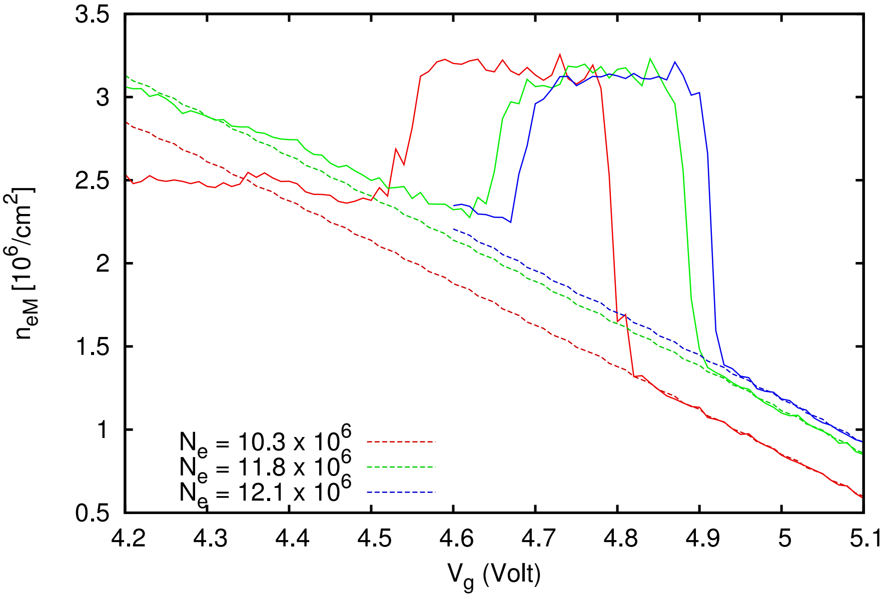

Supp. Fig. 5: Density n_{eM} under microwave irradiation as function of the guard voltage $V_{\rm g}$ obtained from the photocurrent technique at $J = 5.25$ for different $N_{\rm e}$ values. This figure is similar to Fig. 5 in the main text plotted for $J = 6.25$ (solid lines and also showing the appearance of density plateaux under irradiation). Solid and dashed lines represent n_{eM} and $n_{\rm eD}$ respectively.

Supplementary Figure 6: Consistency between compressibility and photo-current measurements at cyclotron resonance.

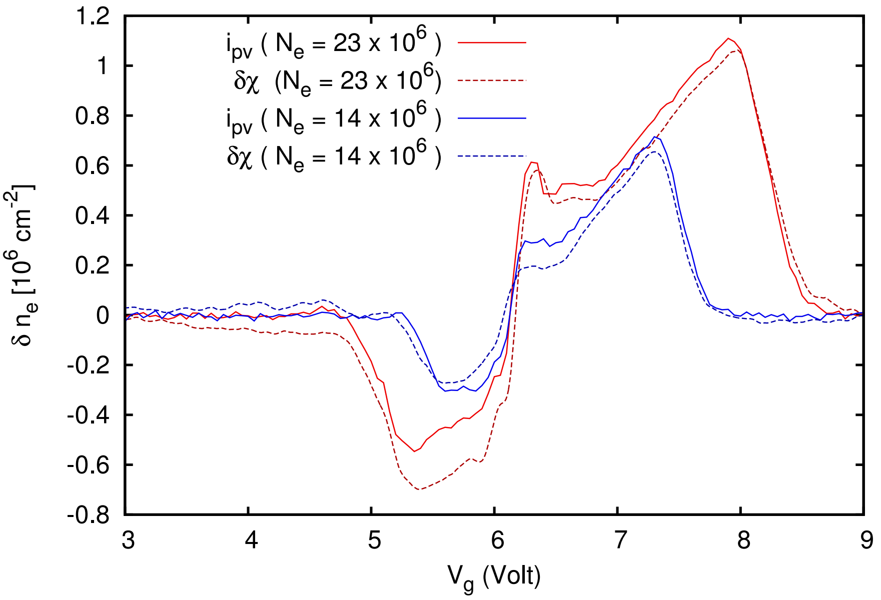

Supp. Fig. 6: Change in the electron density δn_{e} under cyclotron resonance conditions measured through the photocurrent (solid curves obtained from Eq. (19)) and compressibility techniques (dashed curves obtained from Eq. (20)). Cyclotron resonance was excited at a frequency of $\omega = 2\pi \times 14$ GHz corresponding to $B = 0.5$ Tesla, a stronger holding field was applied compared to the case of intersubband excitation $V_{\mathrm{d}} = V_g - V_{tg} = 6$ Volt. The procedure is otherwise identical to that described in the main text in Fig. 4 and 5 in the main text for experiments at intersubband resonance (see discussion in Supplementary note 6).

Supplementary Figure 7: Consistency between compressibility and photo-current measurements at cyclotron resonance.

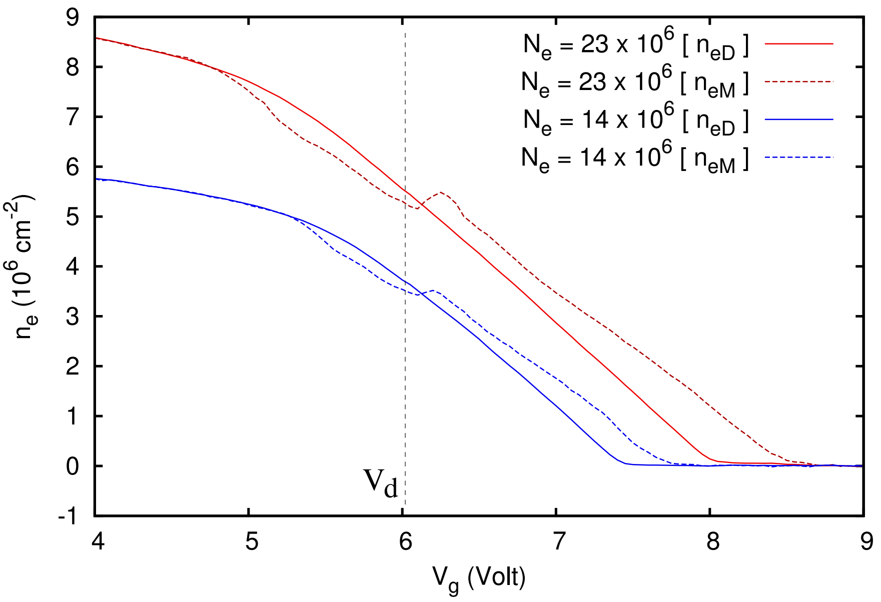

Supp. Fig. 7: Electron density in the dark (solid lines show $n_{\rm eD}$) and under cyclotron resonance conditions (dashed lines for $n_{\rm eM}$) measured by combining photocurrent and dark compressibility measurements as in Fig. 5 from the main text. Density plateaux are not observed in this case even if there is a substantial change in the electronic density. The parameters are the same as those in Fig. 6. The shape of the observed dependence is consistent with heating-induced broadening of the dark $n_{\rm eD}(V_{\rm g})$ dependence. However, the presence of an anomaly around $V_{\rm g} = V_{\rm d}$ suggests that other physical effects may also be important (see also discussion in Supplementary note 6).

Supplementary Figure 8: Consistency between compressibility and photo-current measurements in incompressible phases.

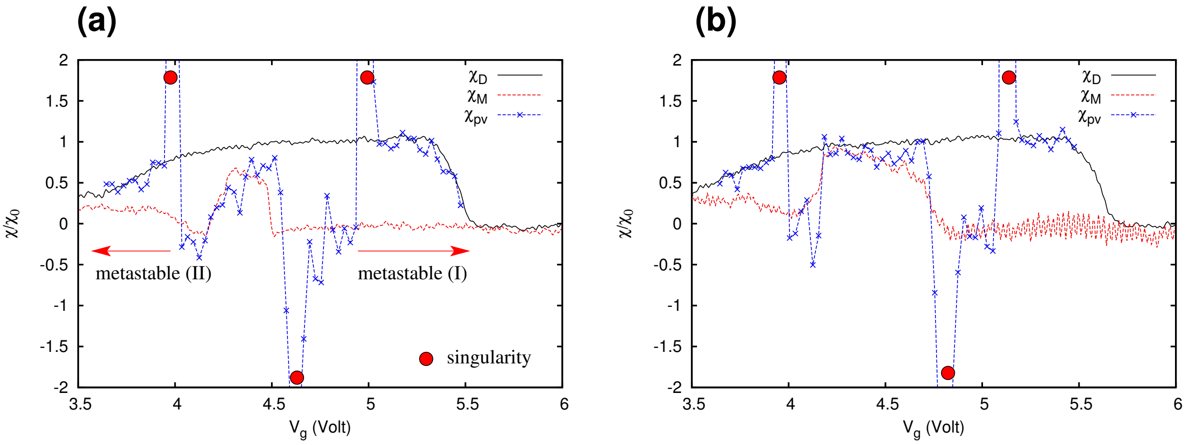

Supp. Fig. 8: The dark and red curves respectively show χ_D and χ_M measured with the low-frequency lock-in technique for $N_{\rm e} = 12.4 \times 10^6$ (**a**) and $N_{\rm e} = 14.5 \times 10^6$ (**b**). The blue curve shows $\chi_{\rm pv}$ calculated by numerical differentiation of the electron density $n_{\rm eM}$ obtained from photocurrent measurements (as a consequence of the differentiation procedure the blue trace is more noisy than the lock-in measurements). The data shown in this figure is taken from the data shown in Figs. 4 and 5 in the main text (see discussion in Supplementary note 7).

Supplementary Figure 9: Consistency between compressibility and photo-current measurements in incompressible phases.

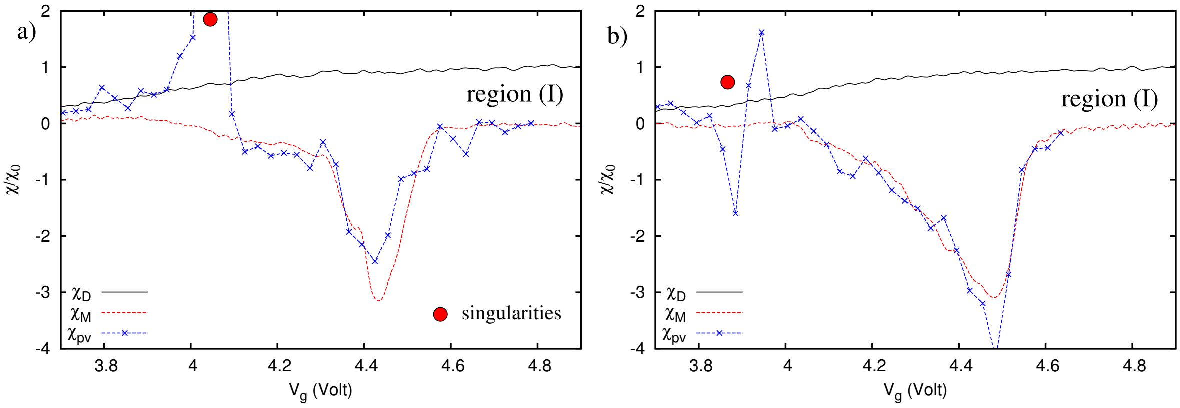

Supp. Fig. 9: Comparison between χ_D, χ_M and $\chi_{\rm pv}$ measured at $N_{\rm e} = 9 \times 10^6$ (left) and $N_{\rm e} = 5.5 \times 10^6$ (right) at $J = 6.25$. This data corresponds to $V_{\rm g}$ scans across the negative compressibility region (see discussion in Supplementary note 7).

Supplementary note 1. On the definition of compressibility.

Our definition of compressibility deviates from the definition of compressibility in the quantum-Hall effect community since the derivative of the density is taken against a gate voltage and not the chemical potential (position of the Fermi level). Such a definition is not suitable in our case since electrons on helium form a non-degenerate electron gas and the Fermi-energy is much smaller than temperature and is therefore not a relevant parameter, instead we have used the electrostatic potential which is controlled by V_g. We note that our experimental technique is otherwise very similar to the capacitive measurements performed on GaAs two-dimensional electron gas to determine the compressibility. For example, except for the dimension of the probe electrode, our measurement geometry is very similar to that in [1] even in terms of the non-local excitation scheme used in their experiment. We would also like to emphasize that as V_g decreases the electron gas is progressively compressed towards the center of the electron cloud, which is the expected behavior in a compressibility experiment. Thus we believe that the use of the term compressibility is fully justified.

Finally our definition of compressibility may seem non-local since it involves the derivative against the potential of the guard electrode V_g instead of the potential of the potential of the central electrode V_d. Actually only the difference $V_\mathrm{d} - V_\mathrm{g}$ is important for the electron distribution profile, thus the two definitions are in principle equivalent. Our motivation for choosing V_g as the main control parameter is that we wanted to keep the perpendicular field constant during our measurements, and it is much easier to fix the potential of the top guard electrode V_{tg} ensuring that $V_{tg} - V_g$ is constant, than to change the potential of the top central electrodes, which are grounded through the current amplifiers.

Supplementary Note 2. Derivation of Eq. (1) in the main text. We will use Δn_e to denote the RMS-oscillation amplitude of the electronic density below the central electrode of diameter R_i induced by AC voltage with amplitude V_{ac} applied to the bottom and top guard electrodes. The oscillation amplitude ΔQ of the charge on the top central electrode then can be expressed as:

$$\Delta Q = \frac{e}{2} \Delta n_\mathrm{e} \pi R_\mathrm{i}^2 \qquad (1)$$

This equation assumes that electrons are distributed midway between top and bottom electrodes separated by distance h. We ensured that this was indeed the case by adjusting the liquid helium level to $h/2$ during the filling of the sample cell using capacitance measurements. Under these conditions it is strictly valid for an homogeneous charge distribution below the disc for which the density gradients are located at least a screening length h away from the electrode edges. This is the case without microwave irradiation since the density gradients are located at $r \simeq R_\mathrm{d} = 1$ cm $> R_\mathrm{i} = 0.7$ cm.

We next consider the effect of a spontaneous density modulation at a wavenumber k superimposed on the average change in the carrier density on the validity of Eq. (1). The influence of wavenumbers for which $kh \gg 1$ will be exponentially suppressed by the screening from the electrodes. The effect of wavenumbers satisfying $kR_\mathrm{i} \gg 1$ will also be reduced by the averaging over the surface of the measuring electrode. At longer wavelengths $kR_\mathrm{i} \ll 1$ the charge density is almost uniform and the error is also expected to be weak. Thus we expect this relation to stay valid for a large range of wave-numbers k. A possible source of charge density oscillation is the fluctuation of the microwave field on the microwave-wavelength scale $\lambda \simeq 2.1$ mm. For this case we have $kh \simeq 7.5$ and $kR_\mathrm{i} \simeq 20.5$ we thus expect the effect of the charge modulations to be strongly suppressed as compared to the changes in the average charge density.

The change in the charge accumulated on the electrodes is related to the current measured with the current amplifier as follows:

$$i_{ac} = -\Delta Q \times 2\pi f_{ac} \qquad (2)$$

where f_{ac} is the modulation frequency. This relation assumes a quasi-static limit where the charges follow the external driving. In a lumped-element approximation this is valid provided the modulation frequency f_{ac} is sufficiently low: $2\pi f_{ac} \sigma_{xx}^{-1} C \ll 1$. Here σ_{xx}^{-1} is the inverse of the longitudinal conductivity of the electron cloud and C is the coupling capacitance. From our measurements using the Sommer-Tanner technique without microwaves we know that under our experimental conditions, with typical electron density $\simeq 2 \times 10^6 \mathrm{cm}^{-2}$ and magnetic field $B \lesssim 1$ Tesla, $2\pi f_{ac} \sigma_{xx}^{-1} C \simeq 1$ at a frequency $f_{ac} \simeq 300$ Hz. We performed our compressibility measurements at $f_{ac} = 2$ Hz, thus the inverse conductivity σ_{xx}^{-1} of the electron cloud can only affect our measurements when σ_{xx} is suppressed by around two orders of magnitude compared with its equilibrium value. As discussed in the main text, the compressibility measurement cannot distinguish between a vanishing conductivity state with $\sigma_{xx} \to 0$ in which carriers do not respond to the external driving owing to their very long response times and an "active" mechanism stabilizing the density at a fixed value independent on the applied electrostatic potentials. We note that the photocurrent experiment (Fig. 5 from the main text) allows us to distinguish between these two scenarios by showing that the cloud reaches the same steady

state density under irradiation independently on the initial density giving a strong argument in favor of an "active" mechanism.

Combining equations Eqs. (1) and (2) we find the compressibility as:

$$\chi = -\frac{\Delta n_{\mathrm{e}}}{\Delta V_{\mathrm{g}}} = \frac{i_{ac}}{e\pi^2 f_{ac} R_{\mathrm{i}}^2 V_{ac}} \tag{3}$$

An implicit assumption here is that $n_{\mathrm{e}}(V_{\mathrm{g}})$ is a continuous well-behaved function. If $n_{\mathrm{e}}(V_{\mathrm{g}})$ has a discontinuity we would theoretically expect a narrow peak smoothed by the lock-in integration-time and instrumental resolution. In the case where an hysteresis loop is present around the discontinuity, the system will stay trapped on the same branch after crossing the discontinuity and the density jump will no longer be correlated with the small voltage modulation V_{ac}. In this case the discontinuity may completely disappear from the compressibility traces. In all cases, we do not expect discontinuities to appear as sharp features in the compressibility measurement.

Supplementary note 3. Derivation of Eq. (2) in the main text.

We denote V_{e} the potential of the electron cloud and assumes that the electron cloud extends to both reservoirs, in the plane condensator approximation the charge densities in the central and guard region are:

$$n_{\mathrm{e}} = -\frac{2\epsilon_0}{eh}\left(2V_{\mathrm{e}} - V_{\mathrm{d}}\right) \tag{4}$$

$$n_{\mathrm{g}} = -\frac{2\epsilon_0}{eh}\left(2V_{\mathrm{e}} - V_{tg} - V_{\mathrm{g}}\right) \tag{5}$$

In these equations we neglected the contribution of the dielectric constant of liquid helium which is close to one within a few percent error.

Subtracting these two equations and keeping only the AC terms we find :

$$\Delta n_{\mathrm{e}} - \Delta n_{\mathrm{g}} = -\frac{4\epsilon_0}{eh} V_{ac} \tag{6}$$

Here we remind readers that the AC potential is applied to both the top and bottom guard electrodes.

Charge conservation in the cloud leads to:

$$S_{\mathrm{d}}\Delta n_{\mathrm{e}} + S_{\mathrm{g}}\Delta n_{\mathrm{g}} = 0 \tag{7}$$

where S_{d} and S_{g} are the surface areas of the central and guard electrodes respectively. We thus obtain the compressibility defined in the text as:

$$\chi_0 = -\frac{dn_{\mathrm{e}}}{dV_{\mathrm{g}}} = -\frac{\Delta n_{\mathrm{e}}}{V_{ac}} = \frac{4\epsilon_0}{eh}\frac{1}{1 + S_{\mathrm{d}}/S_{\mathrm{g}}} \tag{8}$$

For reference, note that we can deduce the potential of the electron cloud with a given total number of electrons N_{e} by inserting Eqs. (4),(5) into:

$$S_{\mathrm{d}} n_{\mathrm{e}} + S_{\mathrm{g}} n_{\mathrm{g}} = N_{\mathrm{e}}, \tag{9}$$

this leads to:

$$V_{\mathrm{e}} = \frac{V_{\mathrm{d}} S_{\mathrm{d}} + S_{\mathrm{g}}(V_{\mathrm{g}} + V_{tg})}{2(S_{\mathrm{d}} + S_{\mathrm{g}})} - \frac{ehN_{\mathrm{e}}}{4\epsilon_0(S_{\mathrm{d}} + S_{\mathrm{g}})} \tag{10}$$

Another way of deriving these equations is by minimization of the charging energy, this derivation also allows us to treat naturally the case where one of the reservoirs is fully depleted under the action of the gate potentials.

Supplementary note 4. Derivation of Eq. (3) in the main text.

This equation connects the change in the electron density under irradiation and the photo-current :

$$\delta n_{\mathrm{e}} = n_{\mathrm{eM}} - n_{\mathrm{eD}} = \frac{2}{e\pi R_{\mathrm{i}}^2}\int i_{\mathrm{pv}}(t)dt \tag{11}$$

where integration is performed over the time interval in which the irradiation is switched off. This equation is derived by combining Eq. (1) with $i_{\mathrm{pv}}(t) = -\Delta Q/\Delta t$ where the ratio is taken in the sense of a time derivative.

The assumptions behind this equation are in practice slightly different from the assumptions behind Eq. (3), indeed since integration is performed over the off phase of the microwave cycle the dynamics of the electrons is known from the dark state properties and the only unknown is the state under microwave irradiation from which relaxation back to equilibrium starts. We thus expect this relation to be less sensitive to short wave-length fluctuations since they will quickly relax back to a homogeneous density without irradiation. Since the two relations correspond to slightly different assumptions on the unknown steady state under irradiation, it is important to ensure that they produce consistent results (this is shown in Figs. 6,8,9)

Supplementary note 5. Derivation of Eq. (4) in the main text.

We assume a more general configuration with fixed n_e and n_g. The electrostatic potentials in the center V_{ed} and in the guard V_{eg} are then different but their values are still given by relations similar to Eqs. (4,5):

$$V_{ed} = \frac{1}{2}\left(V_\mathrm{d} - \frac{eh}{2\epsilon_0} n_\mathrm{e}\right) \tag{12}$$

$$V_{eg} = \frac{1}{2}\left(V_{tg} + V_\mathrm{g} - -\frac{eh}{2\epsilon_0} n_\mathrm{g}\right) \tag{13}$$

The electrostatic energy $U(n_\mathrm{e}, n_\mathrm{g})$ of the system in a plane capacitance approximation is then:

$$U(n_\mathrm{e}, n_\mathrm{g}) = \frac{\epsilon_0}{h}\left[S_\mathrm{d} V_{ed}^2 + S_\mathrm{d}(V_{ed} - V_\mathrm{d})^2 + S_\mathrm{g}(V_{eg} - V_\mathrm{g})^2 + S_\mathrm{g}(V_{eg} - V_{tg})\right] \tag{14}$$

The densities n_e and n_g are related by charge conservation $S_\mathrm{d} n_\mathrm{e} + S_\mathrm{g} n_\mathrm{g} = N_\mathrm{e}$, the electrostatic energy U can thus be viewed as a function of n_e alone $U = U(n_\mathrm{e})$. Moreover an inspection of Eqs.(12,13,14) shows that $U(n_\mathrm{e})$ is a second order polynomial in n_e, for our purposes we will need only the highest order term:

$$U(n_\mathrm{e}) = \frac{e^2 h}{8\epsilon_0} n_\mathrm{e}^2 S_\mathrm{d}\left[1 + \frac{S_\mathrm{d}}{S_\mathrm{g}}\right] + O(n_\mathrm{e}) \tag{15}$$

$$= \frac{e S_\mathrm{d}}{2\chi_0} n_\mathrm{e}^2 + O(n_\mathrm{e}) \tag{16}$$

where we remind that $\chi_0 = \frac{4\epsilon_0}{eh}\frac{1}{1+S_\mathrm{d}/S_\mathrm{g}}$. Since the electrostatic energy is minimal for the dark electron density, we obtain finally :

$$U(n_\mathrm{e}) = \frac{e S_\mathrm{d}}{2\chi_0}(n_\mathrm{e} - n_\mathrm{eD})^2 + \mathrm{const} \tag{17}$$

where the constant is independent on n_e.

Dividing by the total electron number, and using the notation $\delta n_\mathrm{e} = n_\mathrm{eM} - n_\mathrm{eD}$ we obtain the expression for the energy cost of the redistribution per electron which was given without derivation in the main article:

$$\Delta_\mathrm{e} = \frac{U(n_\mathrm{eM}) - U(n_\mathrm{eD})}{N_\mathrm{e}} = \frac{e S_\mathrm{d}}{2\chi_0 N_\mathrm{e}} \delta n_\mathrm{e}^2 \tag{18}$$

Supplementary note 6. Consistency between compressibility and photo-current measurements at cyclotron resonance.

The compressibility under microwave irradiation and the photo-current measurements provide two independent ways of probing the density of the system under microwave irradiation and it is important to verify their consistency. We expect the following relation to hold between the two measurements:

$$\delta n_\mathrm{e} = n_\mathrm{eM} - n_\mathrm{eD} = \frac{2}{e\pi R_\mathrm{i}^2}\int_{Off} i_\mathrm{pv}(t)dt \tag{19}$$

$$= \int_{V_\mathrm{g}}^{\infty} \delta\chi \, dV \tag{20}$$

Here Eq. (19) reproduces Eq. (3) from the main text and Eq. (20), which follows from the integration of $\delta\chi = \chi_M - \chi_\mathrm{d}$ that is the difference between the compressibility $\chi = -\frac{dn_\mathrm{e}}{dV_\mathrm{g}}$ under irradiation and in the dark.

To demonstrate that these two independent measurements indeed lead to the same values of δn_e we performed experiments under cyclotron resonance conditions. As shown recently cyclotron irradiation can significantly heat

the electron system inducing considerable changes in the electron density [2]. This provides us with a model case to confirm the consistency between Eqs. (19) and (20) in a regime where the mechanism driving the electron redistribution appears to be better understood. The results of our experiment are presented in supplementary Fig. 6 and show a good agreement between the two techniques. Supplementary Fig. 7 shows the evolution of the density under irradiation as a function of V_g. The change in the electron density $\delta n_\mathrm{e} \simeq 1 \times 10^6$ cm^{-2} is comparable, but smaller than the changes reported in the main text and the density plateaux do not form under cyclotron resonance conditions.

Supplementary note 7. Consistency between compressibility and photo-current measurements in incompressible phases.

In supplementary figures 6,7, we showed on the example of cyclotron resonance that the compressibility and photo-current measurements are in general consistent with each other. We also verified this for the case of intersubband resonance in regimes where only a small change in the compressibility was observed without leading to the formation of incompressible phases. However, when incompressible states form, the two techniques do not coincide perfectly. For example the voltage span of the incompressible regions is much larger in the compressibility data than in the photocurrent data. Indeed, as seen from Figs. 4 and 5 in the main text region (I) occupies the voltage range $V_\mathrm{g} \in (4\ \mathrm{Volt}, 5\ \mathrm{Volt})$ according to the compressibility data, but the width of the range is only 0.25 Volt according to the photocurrent data for the same number of electrons.

To clarify the origin of this discrepancy we computed the compressibility by numerically differentiating the electron density under irradiation as obtained from the photocurrent measurement; we denote χ_pv as the corresponding value. In supplementary figure 8 we compare χ_pv with the compressibilities χ_D and χ_M measured with the lock-in technique in the dark and under irradiation. In this comparison we used the data shown in the main text at $J = 6.25$ at $N_\mathrm{e} = 12.5 \times 10^6$ and $N_\mathrm{e} = 14.5 \times 10^6$. Except at the singular points that appear due to the differentiation of the abrupt features in Fig. 5 (main text), χ_pv follows the values of χ_D or χ_M switching between the two curves at the singular points located at approximately $V_\mathrm{g} \simeq 4$ and $\simeq 5$ Volt.

This suggests, as described in the main text, that at the switching points the electrostatic energy barrier becomes too high to allow a direct transition to the incompressible state from the dark electron density, collapsing the compressibility χ_pv onto the dark branch χ_D. For χ_M the incompressible behavior can extend over a wider range. Indeed once the system has formed an incompressible state a small change in V_g does not lead to the formation of a large energy barrier. The incompressible state can thus continue to exist as a metastable state that cannot be reached directly from the dark electron density distribution. In this sense, the formation of the incompressible state is strongly hysteretic.

The compressibility measurements under irradiation also show the existence of a region with $\chi_M < 0$ around $n_\mathrm{eD} \simeq n_\mathrm{gD} \simeq 2 \times 10^6$ cm^{-2}. We confirmed that similar negative compressibility values can be obtained independently from the numerical differentiation of the photo-current. The corresponding data is shown on supplementary figure 9. Inside the negative-compressibility region electrons are repelled from the more positive potential effectively behaving as carriers with a positive charge. These experiments show that our two measurement techniques remain consistent even in this exotic state of the electron cloud, highlighting that the discrepancies are indeed due to the presence of singular points in $n_\mathrm{e}(V_\mathrm{g})$ dependence under irradiation. We have not focussed on this regime in the main text since it appears when incompressible region (I) with fixed n_eM and region (II) with fixed n_{gM} merge into the same V_g range. It is thus a consequence of the interaction between the two regions and corresponds to a more complex regime in which the electron system cannot reach a stable final state by increasing the density in one of the reservoirs.

Supplementary references :

[1] S. H. Tessmer, P. I. Glicofridis, R. C. Ashoori, L. S. Levitov and M. R. Melloch, *Subsurface charge accumulation imaging of a quantum Hall liquid*, Nature **392**, 51-54 (1997)

[2] A. O. Badrutdinov, L. V. Abdurakhimov, and D. Konstantinov *Cyclotron resonant photoresponse of a multisubband two-dimensional electron system on liquid helium*, Phys. Rev. B. **90**, 075305 (2014)
